# Antiangiogenic Drugs in NASH: Evidence of a Possible New Therapeutic Approach

**DOI:** 10.3390/ph14100995

**Published:** 2021-09-29

**Authors:** Paola Orlandi, Anna Solini, Marta Banchi, Maurizia Rossana Brunetto, Dania Cioni, Lorenzo Ghiadoni, Guido Bocci

**Affiliations:** 1 Dipartimento di Medicina Clinica e Sperimentale, Università di Pisa, Via Roma 55, 56126 Pisa, Italy; paolaorlandi21@libero.it (P.O.); marta.banchi94@gmail.com (M.B.); maurizia.brunetto@unipi.it (M.R.B.); lorenzo.ghiadoni@unipi.it (L.G.); 2 Dipartimento di Patologia Chirurgica, Medica, Molecolare e dell’Area Critica, Università di Pisa, 56126 Pisa, Italy; anna.solini@unipi.it (A.S.); dania.cioni@unipi.it (D.C.)

**Keywords:** angiogenesis, NAFLD, NASH, VEGF, PlGF, Ang-2, sorafenib, brivanib, ezetimibe, berberine, L1-10, Phyllanthus niruri, ALS-L1023, sitagliptin, losartan

## Abstract

Non-alcoholic fatty liver disease is the most common liver disorder worldwide, and its progressive form non-alcoholic steatohepatitis (NASH) is a growing cause of liver cirrhosis and hepatocellular carcinoma (HCC). Lifestyle changes, which are capable of improving the prognosis, are hard to achieve, whereas a pharmacologic therapy able to combine efficacy and safety is still lacking. Looking at the pathophysiology of various liver diseases, such as NASH, fibrosis, cirrhosis, and HCC, the process of angiogenesis is a key mechanism influencing the disease progression. The relationship between the worsening of chronic liver disease and angiogenesis may suggest a possible use of drugs with antiangiogenic activity as a tool to stop or slow the progression of the disorder. In this review, we highlight the available preclinical data supporting a role of known antiangiogenic drugs (e.g., sorafenib), or phytotherapeutic compounds with multiple mechanism of actions, including also antiangiogenic activities (e.g., berberine), in the treatment of NASH.

## 1. Introduction

Non-alcoholic fatty liver disease (NAFLD) is a progressive liver damage characterized by altered lipid metabolism. If untreated, NAFLD may progress to non-alcoholic steatohepatitis (NASH), cirrhosis, and death [1,2]. Due to the epidemic burden of obesity, type 2 diabetes and metabolic diseases, and NAFLD are likely to become the most common liver disorder in the world [3,4]. NAFLD is a multifactorial, complex disease; its incidence in western countries, although largely underestimated, is increasing. Among predisposing factors, obesity and childhood obesity, sedentary lifestyle, and inadequate dietary habits play a major role. Both environmental and genetic factors are contributing to its development and progression. Recently, a new definition was suggested for NAFLD, namely metabolic dysfunction-associated fatty liver disease (MAFLD) [5]. The prevalence of MAFLD among obese adults worldwide is estimated to be about 50%, and it is relatively higher in men [6]. Abnormalities in several molecular pathways concur to the development of the disease: among these, the Peroxisome Proliferator-Activated Receptors, insulin signaling, Krüppel-Like Factors, p53 signaling, VCAM1, and miRNAs [7]. Insulin resistance leads to an excess delivery of free fatty acid and triglycerides to the liver and a reduced excretion, leading to an intracellular accumulation of triglycerides; additionally, an excess of dietary carbohydrate promotes free fatty acid (FFA) synthesis in the liver. This abnormal hepatic FFA accumulation makes the liver more vulnerable to injury, as represented by the oxidative stress and reactive oxygen species (ROS) production from the mitochondrial respiratory chain, cytochrome P450 FFA metabolism, and hepatic alcohol metabolism. This picture is complicated by obesity, with an abnormal inflammatory state of the adipose tissue and abundant release of inflammatory mediators such as leptin, tumor necrosis factor (TNF)-alpha, and interleukin (IL)-6, which amplify the hepatocyte damage. As a consequence, hepatocytes undergo ballooning, cytoskeletal aggregation, apoptosis, and necrosis. Insulin resistance also accelerates the progression of steatosis to NASH and progressive fibrosis, via sinusoidal collagen deposition [8].

Angiogenesis is a widely described and studied composite process [9,10] that leads to the development of new vessels, resulting from various conditions (including hypoxia) that stimulate the release of angiogenic growth factors, also through the formation of hypoxia-inducible factors (HIFs) [11]. The angiogenic phenomenon physiologically occurs during normal wound healing and also in pathological contexts such as liver disease and tumorigenesis. Thus, numerous antiangiogenic molecules (e.g., the anti-vascular endothelial growth factor (VEGF) monoclonal antibodies bevacizumab) are currently used in the treatment of various cancers, including HCC, according to recent guidelines [12].

**Figure 1:**
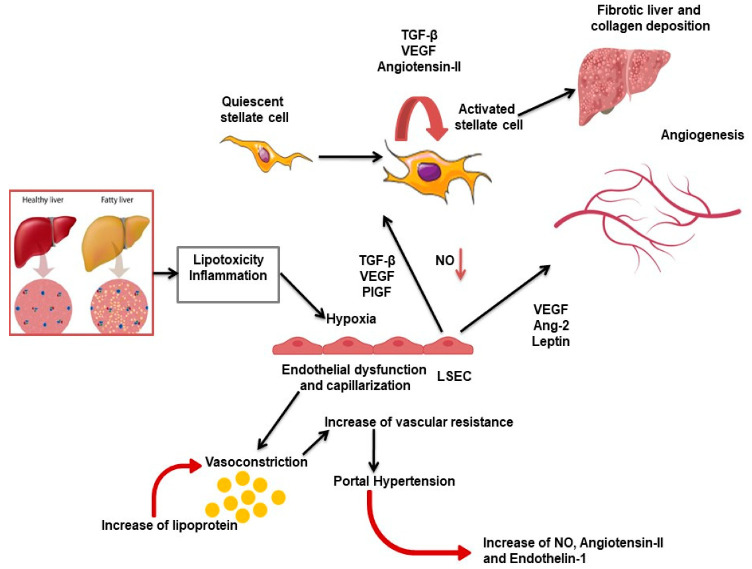
This figure summarizes the common pathways of pathological angiogenesis in NAFLD/NASH discussed in this review. Hepatic steatosis, lipotoxicity, hypoxia, and inflammation induce morphological changes in sinusoidal endothelial cells (LSECs), resulting in endothelial dysfunction and capillarization. LSECs release fibrogenic and angiogenic mediators, while reducing the production of nitric oxide (NO), thus activating hepatic stellate cells (HSCs). At the same time, angiogenic factors released by LSECs and HSC induce hepatic angiogenesis.

Multiple factors trigger angiogenesis in NAFLD, including tissue hypoxia, endothelial dysfunction, hepatic stellate cells (HSC), and inflammation (Figure 1). The role of portal pressure in the early NAFLD stages is particularly important. Indeed, portal hypertension has been demonstrated in patients with NAFLD prior to the development of inflammation or fibrosis and in animal models of steatosis. Recently, van der Graaff et al. [13] hypothesized that structural and dynamic vascular changes in early NAFLD play a role in the progression of the disease by inducing an increased intrahepatic vascular resistance and consequently relative hypoxia for the altered hepatic blood supply in the liver. The hypoxia in the liver tissue causes the capillarization of sinusoids, which are defined by the loss of their fenestrae and the acquisition of a basal membrane [14], and the beginning of the angiogenic process leading to the progression of NAFLD. In particular, HSCs are microcapillary pericytes present in the perisinusoidal space that can be activated after damage, undergoing proliferation and becoming myofibroblasts able to modulate angiogenesis [14]. In the context of chronic liver disease, angiogenesis leads to quantitative changes in the liver vessels with the occurrence of new vessels, but it also involves qualitative changes in the pre-existing vessels, resulting in a process known as vascular remodeling [14]. These qualitative vascular changes include the dedifferentiation of the liver sinusoidal endothelial cells (LSEC), which is a phenomenon also called capillarization [14]. In experimental studies on the angiogenic process during chronic liver disease, it is particularly difficult to discriminate LSEC from vascular endothelial cells in the liver, primarily because not a single marker is completely LSEC-specific, and also because LSECs lose the expression of their canonical markers when they undergo capillarization [14].

In pathological angiogenesis, there is a strong dialogue between different populations of liver cells. This is supported by the concept that the main pro-angiogenic factors such as vascular endothelial growth factor (VEGF), placenta growth factor (PlGF), and platelet-derived growth factor (PDGF) are produced and released by several liver cell types involved in the progression of chronic liver disease (CLD), such as hypoxic hepatocytes, hypoxia-sensitive macrophages, and hepatic myofibroblasts (MF) [15,16,17]. Many experimental studies have reported manifestations of angiogenesis in different NAFLD animal models [18,19,20,21,22]. Indeed, it has been shown that the expression of CD31, the most commonly used marker of endothelial cells, was increased in the liver of mice fed with a high-fat diet (HFD) along with an increase in the expression of VEGFR-2 [19]. Others have reported the induction of CD105 expression, which is a marker of activated endothelial cells acquiring a pro-angiogenic phenotype, in LSECs of mice fed with a diet deficient in methionine and choline (MCD) [21]. Increased VEGF protein has been described in the liver of rats supplied with a choline-depleted amino acid (CDAA) diet [20] and of mice maintained with a MCD diet. In vitro, it has been observed that steatotic hepatocytes produce pro-angiogenic extracellular vesicles [23]. Steatosis induces hypoxia through an increase in lipid metabolism, which enhances oxygen consumption, and by mechanical pressure on the sinusoids. Thus, stellate liver cells, portal myofibroblasts, and macrophages, under these hypoxic conditions, stimulate angiogenesis by secreting VEGF [17]. Pro-angiogenic signals also come from the adipose tissue secreting leptin [24]. Leptin induces an increase in vascular permeability and potentiates VEGF-mediated angiogenesis dose-dependently [25]. Indeed, leptin induces the synthesis of VEGF-A by endothelial cells, and thus the growth of new blood vessels, via the activation of the PI3K/Akt/mTOR/S6 kinase signaling pathway [26].

Hepatic angiogenesis data in animal models of NAFLD and patients suffering from NAFLD suggest a role of this process in the pathogenesis of NAFLD. Angiogenesis is a key step for inflammation and fibrosis [27] in NAFLD. Analyses on hepatic angiogenesis in NAFLD patients are limited in number if compared to those in animal model studies; however, the liver of patients with NAFLD showed increased expression of the endothelial marker von Willebrand factor (vWF), especially in those subjects with advanced fibrosis [28]. Furthermore, in NAFLD patients, a correlation has been observed between vWF and the expression of collagen XV, which is a specific marker of portal myofibroblasts with pro-angiogenic properties by secreting extracellular vesicles containing VEGF [28]. Moreover, NAFLD patients showed significantly higher serum levels of angiopoietin-2 (Ang-2), which is an important protein capable of supporting the angiogenic process in pathologic conditions [29], if compared to those without NAFLD or with simple steatosis [22]. In human steatosis and NASH, the hepatic biopsies revealed an increased content of Ang-2 [22].

This review focuses on the relevance of antiangiogenic agents for the possible treatment of NASH, with specific consideration of different tested drugs, including phytotherapeutic compounds (Table 1), in preclinical experimental settings of liver angiogenesis of NASH models, as well as the possible interactions between these pharmacological approaches to NASH and the prevention of the HCC development.

## 2. Antiangiogenic Drugs and NASH

To date, effective therapeutic strategies have not yet been developed to prevent and treat NASH-mediated cirrhosis and HCC-mediated liver cirrhosis. Clinical studies have demonstrated that a high-fat diet is closely related to the development of NASH [30]. Moreover, scientific literature demonstrates that cholesterol levels are closely associated with VEGF, which is a key factor promoting HCC [31]. NAFLD may result in a variety of liver diseases such as liver fibrosis, cirrhosis, and HCC [32]. Antifibrotic therapies have been studied in order to reverse liver fibrosis [33]. Various compounds have been tested for their antifibrotic mechanism of action, such as the degradation of the extracellular matrix, the antioxidant activity, the reduction of inflammation, and the inhibition of the activation of HSCs [34]. Angiogenesis is a key step for the development of fibrosis and, thus, among the molecules tested for their antifibrotic activity, also the antiangiogenic drugs have been included.

### 2.1. Sorafenib

Sorafenib, a tyrosine and serine/threonine kinase inhibitor, is an FDA-approved first-line therapy for advanced HCC [35,36]. It is active also on other human tumors such as advanced renal cell carcinoma [37] and differentiated thyroid carcinoma [38], leading to a reduction in tumor angiogenesis. Indeed, sorafenib inhibits the phosphorylation of various targets present in the signaling pathways of both tumor cells (i.e., CRAF, BRAF, V600E BRAF, c-KIT, and FLT-3) and in the tumor-endothelial cells (i.e., CRAF, VEGFR-2, VEGFR-3, and PDGFR-β) [39,40,41]. Sorafenib showed antiangiogenic and antifibrotic activity on HSCs and hepatic endothelial cells in preclinical models [42,43,44,45]. Indeed, several experimental studies have demonstrated that after treatment with sorafenib, the number of activated HSCs was reduced [46], the intrahepatic fibrosis and inflammation decreased, and the process of angiogenesis diminished [47]. These effects led to the suppression of collagen accumulation, with a significant decrease in HSC number [45]. Yang et al. [48] have also shown that in addition to the decreasing fibrosis, portal hypertension, and angiogenesis, the anti-VEGFR action of sorafenib improves the hepatic blood flow and inhibits the activation of leukocytes, the accumulation of splanchnic blood, and the formation ascites in NASH cirrhotic rats. Moreover, in a preclinical rat model with NASH [44], sorafenib was able to reduce collagen, increase matrix metalloproteinase (MMP) mRNA levels, and decrease the protein expression of tissue metallopeptidase inhibitor-1 (TIMP-1) as well as the pro-inflammatory interleukins (IL)-6 and 10 [44]. Another in vivo study by Jian et al. [49] showed that sorafenib administered at low doses decreased hepatic steatosis, inflammation, and fibrosis, thanks to the activation of the protein kinase 5’ by adenosine monophosphate (AMPK). Interestingly, in this study, performed in mice and monkeys, the researchers evaluated the efficacy of this new low-dose modality of sorafenib treatment to prevent the early stage of hepatocarcinoma using a mouse model of NASH-HCC [49]. Sorafenib was able to effectively block the onset of HCC at a dose equivalent to one-tenth of the current clinical application, greatly improving fatty liver, inflammation, and fibrosis, which are typical manifestations of NASH without causing any detectable adverse events [49]. Considering that fibrosis and cirrhosis determine an increased risk of developing HCC [50,51,52], the use of sorafenib, as can be seen from the numerous data obtained in vitro and in vivo, could represent an effective chemopreventive pharmacological tool for the development of hepatocarcinoma. In fact, the treatment with sorafenib during the evolution of NASH could block the early stages of hepatocarcinogenesis by decreasing fibrosis or directly blocking pre-malignant liver lesions, as demonstrated in a rodent model [53].

### 2.2. Brivanib

Brivanib is a VEGFR-2 and a fibroblastic growth factor (FGFR) tyrosine kinase inhibitor with strong antifibrotic [54] and antineoplastic preclinical activity [55]. It specifically and strongly binds to human VEGFR-2 expressed almost exclusively on vascular endothelial cells. The blockade of VEGFR-2 by brivanib inhibits the migration and proliferation of VEGF-stimulated endothelial cells, resulting in the arrest of tumor angiogenesis [56]. This compound has shown some antitumor activity when administered to patients with hepatocellular carcinoma refractory to other antiangiogenic therapies [55]; however, there are few data on its effects on cirrhosis. In 2014, Nakamura et al. published a report on brivanib’s ability to decrease the hepatic fibrosis in vivo and the HSC activation in vitro through the inhibition of FGF, VEGF, and PDGF signaling [57]. Indeed, after in vivo induction of liver fibrosis with different modalities such as bile duct ligation, or the treatment with chronic carbon tetrachloride or thioacetamide, the mice administered with brivanib resulted in a decreased liver fibrosis and a reduced expression of collagen Iα1 and α-smooth muscle actin in the liver [57]. Brivanib also decreased HSC viability and blocked the PDGFBB-induced phosphorylation of its receptor [57]. In the same year, Yang et al. [48] studied the effects of a 2-week treatment with brivanib (or sorafenib) on cirrhotic rats with NASH. Significant decreases in plasma levels of VEGF, FGF, PDGF, liver tumor necrosis factor (TNFα), IL-1b, IL-6, and IL-17 were observed in NASH-cirrhotic plus brivanib and NASH-cirrhotic plus sorafenib rats during the treatment period if compared to those treated with vehicle alone [48]. Moreover, a general improvement in hepatic blood flow, as well as a decrease in hepatic neovascularization and portal hypertension, were reported during the treatment period; the inhibition of the inflammation, portal fibrosis, and ascites formation was also described [48].

### 2.3. Anti-VEGFR-2 Antibody

Coulon et al. [21] evaluated the role of angiogenesis in two mouse models affected by NASH. The effect of preventive and therapeutic antiangiogenic treatment was observed in a diet-induced mouse model of NASH. This study demonstrated that angiogenesis is induced during the pathophysiology of NASH. In fact, in this experimental setting, the onset of NASH was usually accompanied by a significant increase in inflammatory and angiogenic factors. Among the main factors involved in pathological angiogenesis in various chronic liver diseases [27], the authors highlighted the role of both VEGF and the placental growth factor (PlGF) on NASH progression as new targets for treating or preventing the disease [21]. Interestingly, mice with NASH treated with an anti-VEGFR-2 antibody showed a better and more organized vascularization if compared to untreated mice. Moreover, primary hepatocytes treated with an anti-VEGFR-2 antibody were also able to incorporate a much lower amount of lipids [21]. Finally, the researchers showed that a treatment with an anti-VEGFR-2 antibody had a preventive and therapeutic role in decreasing steatosis and inflammation in the liver of mice with NASH. Conversely, an anti-PlGF antibody did not significantly improve hepatic histology and liver fibrosis [21].

### 2.4. Ang2–Tie2 Interaction Inhibitors: L1-10

Conditions such as tumor and inflammation lead to pathological angiogenesis, and various angiogenic factors are involved in this complex process. One such angiogenic factor is Ang-2 [29]. Indeed, even though Ang-2 physiologically inhibits angiogenesis, it is overexpressed in diseases such as cancer, and its ability to induce angiogenesis in this case has been demonstrated [58]. Both Ang-1 and Ang-2 bind to specific tyrosine kinase receptors named Tie receptors (Tie1 and Tie2), which are almost exclusively expressed on the surfaces of endothelial cells [29]. It is believed that Tie1 is an orphan receptor without a ligand that is able to modulate the angiopoietins signaling via Tie2 [59]. In basal and non-inflammatory conditions, Ang-1 controls normal vascularization by binding to the Tie1/Tie2 complex. In inflammatory processes, overexpressed Ang-2 competes for receptor binding, acting as an antagonist of Ang-1 and leading to a destabilization of blood vessels and to vessel remodeling [60]. Therefore, in pro-inflammatory conditions, the Ang-2–Tie1/Tie2 receptor complex association promotes abnormal vascular remodeling [61].

An increase in serum Ang-2 was observed in an animal model of HCC-NASH (i.e., neonatal streptozotocin, STAM mice) and fed for 16 weeks on a Western diet [62]. Lefere et al. [22] showed that in NASH patients, the serum levels of Ang-2 increased directly and proportionally to the degree of inflammation, steatosis, swelling, and histological alteration, but not to the degree of fibrosis. In this paper, the correlation between Ang-2 and CD34 expression, a marker of neoangiogenesis, in liver histological sections was also highlighted. Moreover, hepatic vascular endothelial cells under inflammatory conditions secreted Ang-2. Lefere et al. studied the Tie1/Tie2 receptor complex and its angiopoietin ligands also in a murine non-alcoholic fatty liver disease model [22], and they confirmed that in mice fed MCD, serum levels of Ang-2 are increased. L1-10 is a peptide antibody and an Ang-2 selective inhibitor that showed 1000-fold inhibitory selectivity for Ang-2 over Ang-1 [63]. L1-10 abolished the in vitro binding affinity between Ang2 and Tie2 in a dose-dependent manner [64]. Interestingly, the administration of L1-10 reduced the liver inflammation, ballooning, and fibrosis in MCD-fed mice but did not change the degree of steatosis. The authors also demonstrated that L1-10 treatment reduced angiogenic signaling from cultured endothelial cells after LPS stimulation, confirming their observations that L1-10 reduces neoangiogenesis primarily through endothelial cell signaling [22]. However, to date, there are no clinical trials that have used L1-10, but trebananib, an Ang-2 neutralizing peptide antibody, has been already tested as an additional therapy in advanced cancer diseases [65,66], revealing a good toxicity profile in patients, although the most frequent side effects were edema and ascites [65,66]. Unfortunately, in patients with advanced HCC, the introduction of trebenanib in addition to the standard treatment represented by sorafenib did not result in any improvement of progression-free survival [67].

## 3. Drugs with Antiangiogenic Activities and NASH

### 3.1. Ezetimibe

The growth of some tumors, such as breast cancer [68] and prostate cancer [69], may be favored by excess cholesterol, which promotes angiogenesis. In NASH, an increase in leptin-mediated angiogenesis was observed [20], and the inhibition of the neovascularization process decreases the severity of the disease [21]. Moreover, high cholesterol levels induced angiogenesis in hepatocyte specific phosphatase and tensin-deficient (Pten) mice (*Pten^Δhep^* mice) following the HF diet [70]. Increased cholesterol not only causes liver damage but also induces Kupffer cells to express VEGF [71].

Ezetimibe, a specific cholesterol uptake-blocking drug, has been shown to inhibit angiogenesis and retard prostate cancer growth [69]. Based on this premises, Miura et al. [72] evaluated the effects of ezetimibe in the experimental model of *Pten^Δhep^* mice, which develops HCC after steatohepatitis [73]. Interestingly, in this model of HCC related to steatohepatitis, mice fed with a high-fat diet showed a concomitant, significant increase in cholesterol and VEGF serum levels. Ezetimibe was able to block the growth of HCC by inhibiting cholesterol-mediated angiogenesis in *Pten^Δhep^* mice with hypercholesterolemia; by contrast, it did not affect angiogenesis in *Pten^Δhep^* mice fed with a standard diet, in which cholesterol elevation was low [72].

### 3.2. Losartan and Sitagliptin

In CLD, the renin–angiotensin system plays a crucial role [74]. In fact, the block of angiotensin II (AT-II) signal transduction through the AT-II type 1 receptor (AT1R) is able to inhibit hepatic fibrogenesis and, at the same time, suppress the activation of hepatic stellate cells [75]. Losartan, by blocking the renin–angiotensin system, inhibited hepatocarcinogenesis and the growth of HCC [76,77]. Sitagliptin, a selective inhibitor of dipeptidyl peptidase-4 (DPP-4I), is a drug used to treat type 2 diabetes mellitus that is suggested to be useful in NAFLD [78]. The expression of DPP-4 is elevated in many cell populations, including endothelial cells, and it is implicated in inflammation and tumorigenesis [79].

Okura et al. [80] described the chemopreventive effects of losartan and sitagliptin combination on hepatic stellate cell activation, angiogenesis, and oxidative stress, which are key steps in NASH progression, in a rat model of NASH. The authors showed that losartan plus sitagliptin were able to reduce hepatic fibrogenesis and carcinogenesis caused by the CDAA diet, almost in parallel with the suppression of neovascularization and oxidative stress [80]. The inhibitory effect of losartan plus sitagliptin on hepatocarcinogenesis was mediated by the inhibition of endothelial cell tube formation, not by a direct action on cell proliferation of endothelial and HCC cells [80]. By suppressing hepatic neoangiogenesis caused by VEGF, the chemopreventive effect on experimental hepatocarcinogenesis mediated by sitagliptin plus losartan could be achieved in synergy, reaching the control of hepatic fibrogenesis and the inhibition of carcinogenesis [80].

## 4. Phytotherapeutic Compounds with Antiangiogenic Properties and NASH

### 4.1. Berberine

Berberine is an isoquinoloin alkaloid present in several plants of the Berberidaceae family. The therapeutic use of berberine as a remedy for acute gastroenteritis and diseases of the digestive tract [81] comes from Chinese medicine. Berberine has shown anticancer properties in preclinical models [82]. In fact, it appears to be effective on liver, colon, lung, breast cancer, melanoma, neuroblastoma, and other cancer cells [83,84,85]. Moreover, berberine has been shown to be an effective antiangiogenic compound by decreasing the expression of VEGF and HIF-1alpha [86].

A NASH–HCC mouse model has been developed by Fujii et al. [87] using a streptozotocin (STZ) injection associated with a high-fat, high-cholesterol (HFHC) diet. This mouse model mimics the entire pathological process from fatty liver, steatohepatitis, and fibrosis to HCC [87]. Luo et al. [88] investigated the mechanism of action and the therapeutic potential of berberine in this particular model of NASH–HCC. Mice were treated for three months with berberine (250 mg/kg daily) by gavage, as previously described [89,90]. As expected, the HFHC diet combined with the STZ injection induced the growth of tumors in the mouse liver of the control group, whereas in the berberine-treated mice liver, tumorigenesis was attenuated, with the development of very few tumors [88]. Interestingly, in STZ–HFHC mice, the berberine compound reduced the microvascular density (MVD). Moreover, in STZ–HFHC mice, the increased expression of CD31 and VEGF was suppressed by berberine, confirming its potential antiangiogenic characteristic. Even more, berberine significantly reduced the levels of liver enzymes, glucose, high-density lipoprotein, low-density lipoprotein and total cholesterol, as well as the expression of IL-6, IL-1β, MCP-1, and TNF-α demonstrating its positive metabolic and anti-inflammatory effects [88].

### 4.2. Phyllanthus Niruri

Phyllanthus niruri is a small annual herbaceous plant native to the Amazon rainforest and other tropical areas, including Southeast Asia, South India, and China. It is a medicinal plant widespread throughout the tropical and subtropical world and widely present in the coastal areas of India where it has been used in the Ayurvedic medicine for more than 2000 years [91]. Its extract is an excellent antioxidant, a good hepatoprotector, and lowers blood lipid levels [92,93], at least in preclinical models.

In the study by Zazour et al. [94], the antiangiogenic properties of the standardized 50% methanolic extract of Phyllanthus niruri (50% ME of P. niruri) were investigated in a model of NAFLD in Sprague–Dawley rats. In vitro and in vivo tests were performed to evaluate the inhibition of endothelial cell migration, tube formation, and VEGF activity by 50% ME of P. niruri without any cytotoxic effect. Indeed, a significant antiangiogenic effect was obtained by inhibiting the development of microvessels in the rat aorta model, decreasing the migration and the differentiation of endothelial cells [94]. Above all, 50% ME of P. niruri effectively attenuated NAFLD, with a preventive effect on fibrosis, which was accompanied by the inhibition of VEGF production [94]. In fact, the block of VEGF, which activates HSC, is one of the mechanisms of inhibition of fibrosis as previously shown by Coulon et al. [11].

### 4.3. ALS-L1023

The growth and increase in adipose tissue, as well as the formation of neoplastic tissue, are thought to be dependent on the angiogenic process [95]. The onset and progression of NAFLD is related to the presence of visceral adipose tissue (VAT); thus, Kim et al. [96] argued that NAFLD caused by obesity could be blocked by inhibiting angiogenesis. The same authors have previously shown that the antiangiogenic herbal extract Ob-X was able to significantly reduce adipose tissue and suppress obesity by inhibiting angiogenesis [97]. Indeed, other inhibitors of angiogenesis (such as TNP-470) significantly reduced body weight and fat mass [98,99] in mice, indicating a role of angiogenesis in the growth of fat tissue. For these reasons, Kim et al. [96] investigated a new phytotherapic remedy called ALS-L1023, the active part of an organic extract of lemon balm leaves (Melissa officinalis L.), which is endowed with antiangiogenic activity and was previously discovered by Park et al. and Woo et al. [100,101]. The antiangiogenic effects of ALS-L1023 in relation to visceral obesity and NAFLD were evaluated in high-fat C57BL/6J mice (fed with HFD). ALS-L1023 extract had actually a significant antiangiogenic action because in HFD-ALS-L1023- treated mice, compared to only HFD mice, there was a decrease in the expression of VEGF and an increase in thrombospondin-1 (TSP-1) [96], which is a well-known endogenous inhibitor of angiogenesis [102].

## 5. Conclusions

Only a small percentage 20–30% of patients with NAFLD develop NASH, fibrosis, cirrhosis, and hepatocarcinoma [103]. About 7% of cirrhosis associated with NAFLD will evolve in hepatocellular carcinoma within 10 years [103]. Currently, weight loss and lifestyle change through diet and exercise is recommended as a first-line therapy [104,105]. However, long-term compliance with lifestyle changes is difficult to achieve and maintain in the target population, and a standardized drug therapy for these diseases is still lacking. As a result, a major unmet need for a new drug to treat NASH and reverse liver fibrosis exists.

In recent years, numerous clinical studies are underway for the evaluation of new drugs that should act on the pathogenetic mechanisms (insulin resistance, alteration of lipid metabolism) that lead to NASH. As an example, in the recent review article by Dehnavi et al. [106], we reported the published data about the peculiar characteristic of statins to reduce hepatic lipid accumulation and thus to have a therapeutic use in NAFLD. In particular, atorvastatin and fluvastatin promote the AMPK signaling pathway, inhibiting the acetyl CoA carboxylase, which is a key enzyme for lipogenesis, and blocking the fat accumulation in the hepatocytes. Thus, statins have hepato-protective effects through the regulation of AMPK signaling [106]. In our review, we analyze the antiangiogenic drugs that may play a major role in the pathogenetic mechanism that leads to the progression of liver damage. However, the use of these antiangiogenic molecules does not exclude therapeutic interventions aimed at blocking the etiopathogenetic mechanisms; indeed, these drugs could have an adjuvant role in particular in the most advanced forms of damage, when fibrosis is greater.

Antiangiogenic drugs, and in particular sorafenib, may impact negatively on type 2 diabetes. However, Imarisio et al. [107] have published an interesting clinical trial on patients with advanced HCC or metastatic renal cell carcinoma and comorbid diabetes mellitus or prediabetes. All these patients were treated with 400 mg sorafenib twice daily for approximately 8 months (a standard anticancer dosage). The authors concluded that sorafenib has the potential to be a feasible and safe treatment option for these patients. Moreover, Makol et al. [108] observed that the percentage of HCC patients responding to sorafenib was higher in type 2 diabetes group and that after three months of treatment, their glycemia decreased significantly, which was probably due to the enhancement of glycolysis by sorafenib [109].

Encouraging results suggest that low-dose sorafenib could be used for the treatment of NASH and the co-treatment with brivanib may offer the possibility of using these anti-VEGFR drugs over a long term. Moreover, due to Ang-2 contributing to the progression of NASH, it emerged that Ang-2 inhibitors, such as L1-10, may be effective in the prevention and resolution of steatohepatitis by inhibiting pathological vascular growth and endothelial cell dysfunction. Finally, other commonly used drugs, such as ezetimibe, or phytotherapeutic remedies such as berberine or ALS-L1023, may be effective in inhibiting the progression of disease through the block of the angiogenic process and the reduced VEGF secretion.

There is increasing evidence that neovascularization is a key element in the progression of NAFLD (Figure 1). The formation of new blood vessels in chronic liver disease is linked to the advancement of fibrosis, indicating a close interplay between LSECs and HSCs. In normal liver, sinusoidal homeostasis depends on low-level release of VEGF by hepatocytes, helping LSECs to remain differentiated and to generate nitric oxide, which inhibits the activation of HSCs [110]. Although VEGF is an essential regulator in maintaining LSEC differentiation [111], in NAFLD, hepatocytes and nonparenchymal liver cells increased VEGF production, mediating both pro-fibrogenic and pro-angiogenic signals, supported by HIF activation in hypoxic areas [110]. Indeed, serum VEGF levels of patients with steatosis and steatohepatitis are higher compared to healthy controls [110]. In this perspective, low-dose TKIs may help to restore physiologic levels of VEGF signal, maintaining the physiologic functions on LSECs.

Some antiangiogenic drugs (i.e., sorafenib and brivanib) have been shown important adverse events at the standard doses administered to cancer patients. Although the eventual experimental dosages of sorafenib or brivanib in NASH could be lower and safer compared to the one administered in the oncology field, caution should be used because these drugs can deteriorate the prognosis of NASH patients with cardiovascular morbidity. However, severe adverse events due to high plasma concentrations of TKIs may be also addressed by the application of TDM-guided dosing, ensuring levels within the therapeutic window [112]. Particularly relevant on this issue is the very recent meta-analysis by Hou et al. performed to identify the potential cardiotoxicity risks of VEGFR–TKIs in patients with solid tumors [113]. These authors concluded that among the VEGFR–TKIs, lenvatinib and vandetanib revealed the highest possibility to provoke cardiovascular events and hypertension, followed by cabozantinib, axitinib, pazopanib, sorafenib, sunitinib, regorafenib, and nintedanib. Although sorafenib has been shown to cause these important adverse reactions, the risk of a cardiovascular event due to this drug is significantly lower than that of many other VEGFR-TKIs. Interestingly, regorafenib and nintedanib do not exhibit an increased risk of cardiovascular incidents and therefore may represent a valid experimental alternative in the treatment of NASH or NAFLD to sorafenib and other antiangiogenic drugs. Moreover, data from the randomized phase III BRISK-FL study [114] showed that in patients with HCC, the most frequent grade 3/4 adverse events related to the administration of sorafenib and brivanib were hyponatremia, fatigue, hand–foot–skin reaction, and hypertension (5% and 13%, respectively).

Another important aspect of the eventual use of sorafenib in NASH is the potential pharmacological interactions with antidiabetic or antihypertensive drugs that are commonly prescribed in NASH patients. Recently, Karbownik et al. [115] investigated the pharmacokinetic interactions between sorafenib and metformin or atorvastatin in a rat model. The concomitant administration of sorafenib and metformin increases the clearance of sorafenib in rats, which results in a significantly lower sorafenib half-life (16.3 vs. 21.9 h). Moreover, metformin also significantly decreased the area under the curve (AUC) of sorafenib. On the contrary, sorafenib did not statistically influence the pharmacokinetic parameters of metformin. Sorafenib is mainly metabolized both via CYP3A4 isoform and UGT1A9. Felodipine, an anti-hypertensive agent that is exclusively a CYP3A4 substrate, has been demonstrated to cause changes in sorafenib pharmacokinetic parameters in an 80-year-old HCC patient with hypertension [116]. Indeed, after 30 days of co-treatment, sorafenib plasma concentration was three-fold greater, and although the hypertension was well controlled, the patient experienced a grade-3 anorexia. Since hypertension is a possible adverse drug reaction of sorafenib, oncologists and endocrinologists should be aware of this possible interaction.

Although there are consistent data on the use of the antiangiogenic approach for the treatment of NASH with several tested drugs, unfortunately, no dedicated, randomized phase III clinical trials have been planned and performed to give a clinical answer to the possible contribution of the antiangiogenic drugs into NASH therapy. Indeed, the “hepatological world” looks at sorafenib and to other antiangiogenic compounds exclusively as drugs for the patient with advanced cancer (therefore in very advanced clinical contexts). Therefore, the hypothesis of their use alone or in combination with other drugs in NASH patients, even if in very small doses, must be introduced in the researcher and clinician communities, although with caution due to the possible cardiovascular toxicities.

## Figures and Tables

**Table 1:** Antiangiogenic treatments in animal models of NASH and NAFLD.

Antiangiogenic Treatment	Animal Model	Results	Reference
Sorafenib 40 mg/kg, 20 mg/kg, 5 mg/kg, and 1 mg/kg, orally	Male Sprague–Dawley and Wistar rats	Decreased liver fibrosis, reduced HSC proliferation, downregulation of cyclin D1 and cyclin-dependent kinase 4 and inhibition of the ERK and Akt phosphorylation.	Wang et al., 2010
Sorafenib 4 mg/kg intragastrically once a day for four weeks	C57BL/6 (B6) mice	Attenuation of CCl4-induced chronic liver injury and fibrosis.	Deng et al., 2013
Sorafenib 1.25, 5 or 7 mg/kg/day orally	Male Sprague–Dawley rats	Significant inhibition of liver fibrosis when administered concurrently with TAA. No significant effect on fibrosis when administered after established cirrhosis.	Hong et al., 2013
Sorafenib 2.5 mg/kg/day, orally	Adult Sprague–Dawley rats	Restoration of mitochondrial function and reduction of collagen deposition in a NASH model. Upregulation of PGC1a and MMP9; reduction of TIMP1, TIMP2 mRNA, and IL-6, IL-10 protein.	Stefano et al., 2015
Sorafenib 10, 15, and 30 mg/kg/ every 2 days	Male C57BL/6J mice	Significant reduction of HCC incidence and size in a model of NASH. Suppression of the pathological features of NASH, including hepatic steatosis, inflammation, and fibrosis.	Jian et al., 2020
Sorafenib 10 mg/kg/day orally for 2 weeks	Male albino rats	Prevention of neoplastic changes in the liver with a decrease in size of hepatocellular foci.	El-Ashmawy et al., 2017
Anti-VEGFR-2 (40 mg/kg i.p.) and Anti-PlGF (25mg/kg i.p.) antibodies	Ten-week-old C57BL/6 and homozygous db/db female mice	Prevention of NASH progression by decreasing steatosis and inflammation (anti-VEGFR-2). No effect of anti-PlGF on liver histology. Improvement of the liver vasculature by anti-VEGFR-2.	Coulon et al., 2013
Brivanib (3 mg/kg/day), sorafenib (5 mg/kg/day), orally	Male Wistar rats	Significant decrease in plasma VEGF, FGF, PDGF, hepatic TNFα, IL-1b, IL-6, IL-17; decrease in hepatic leucocytes recruitment, microvascular density and hydroxyproline content; increased hepatic blood flow in NASH-cirrhotic rats.	Yang et al., 2014
Ezetimibe 50 mg/kg orally	Pten^Δhep^ mice (C57BL/6 background)	Blockade of the development of HCC by inhibiting cholesterol-mediated angiogenesis in Pten^Δhep^ mice with hypercholesterolemia. Conversely, no inhibition of angiogenesis in Pten^Δhep^ mice fed with the standard diet	Miura et al., 2019
Berberine 250 mg/kg/day orally	C57BL/6J mice	Suppression of genes related to lipogenesis, inflammation, fibrosis, and angiogenesis.	Luo et al., 2019
L1-10 4 mg/kg i.p. three-times weekly	C57BL/6 mice	Reduction of liver inflammation, balloon, and fibrosis in MCD-fed mice; reduction of angiogenic signaling in cultured endothelial cells.	Lefere et al., 2019
50% ME of Phyllanthus niruri (1000 mg/kg orally).	Sprague-Dawley rats	Attenuation of NAFLD with a preventive effect on fibrosis accompanied by the inhibition of VEGF production.	Al Zarzour et al., 2018
ALS-L1023 (0.8%, *w*/*w*; orally)	C57BL/6J mice	Suppression of steatosis, infiltration of inflammatory cells, and accumulation of collagen in livers. Fewer CD68-positive macrophage numbers and lower expression of inflammatory cytokines.	Kim et al., 2017
Sitagliptin 150 mg/kg/day, losartan 30 mg/kg/day orally, alone and in combination	Fischer 344 rats	Combined treatment suppressed hepatic fibrogenesis and carcinogenesis, with the suppression of HSC activation, neovascularization, and oxidative stress.	Okura et al., 2017

## Data Availability

Data sharing not applicable.
